# Supplementary material for: Uninterrupted or Minimally Interrupted Direct Oral Anticoagulant Therapy is a Safe Alternative to Vitamin K Antagonists in Patients Undergoing Catheter Ablation for Atrial Fibrillation: An Updated Meta-Analysis
Source: J Clin Med. 2020 Sep 24;9(10):3073. doi: 10.3390/jcm9103073 (PMC7598585; doi:10.3390/jcm9103073)
Supplement: Supplementary file 1 [file jcm-09-03073-s001.zip › Supplementary.pdf]

Supplementary material

List of Supplementary Figures and Tables

Table S1. Assessment of bias using the Newcastle–Ottawa Scale

Table S2. Assessment of bias in randomized clinical trials using the Cochrane Collaboration Tool

Table S3. Detailed baseline characteristics

Table S4. Results of Leave-One-Out analysis in the uninterrupted and minimally interrupted treatment group, major bleeding

Table S5: Results of Leave-One-Out analysis in the RCT group, composite endpoint

Figure S1. Outcome of RCTs: Stroke and TIA

Figure S2. Outcome of RCTs: Major Bleeding

Figure S3. Composite endpoint of RCTs

Figure S4. Minimally interrupted and uninterrupted therapy outcomes: Minor bleeding

Figure S5 Interrupted therapy outcome: Stroke and TIA

Figure S6 Interrupted therapy outcome: Major bleeding

Figure S7. Interrupted therapy outcome: Minor bleeding

Figure S8. Baseline characteristics of the included studies

- **Figure S8.1** Age
- **Figure S8.2** Males
- **Figure S8.3** BMI
- **Figure S8.4** CHADSVASc
- **Figure S8.5** HASBLED

Table S1: Assessment of bias using the Newcastle-Ottawa Scale

AUTHOR & DATE	Selection				Comparability	Outcome			TOTAL
	1	2	3	4	5	6	7	8	
Armbruster 2014	1	1	1	1	2	1	90d	1	8
Arshad 2014	1	1	1	1	2	1	4-6w	1	8
Aso 2014	1	1	1	1	0	1	?	0	5
Bassiouny 2013	1	1	1	1	1	1	30d	0	6
Brinkmeier-Theofanopoulou 2018	1	1	1	1	2	1	48h	0	7
Di Biase 2015	1	1	1	1	2	1	30d	1	8
Dillier 2014	1	1	1	1	2	1	3m	1	8
Heide 2018	1	1	1	1	2	1	30d	1	8
Ichiki 2013	1	1	1	1	2	1	?	0	7
Kaiser 2013	1	1	1	1	2	1	90d	0	7
Kaseno 2012	1	1	1	1	2	1	4w	1	8
Khan 2013	1	1	1	1	0	1	?	0	5
Kim 2013	1	1	1	1	2	1	3m	1	8
Kojima 2018	1	1	1	1	2	1	?	0	7
Lakkireddy 2012	1	1	1	1	2	1	30d	1	8
Lakkireddy 2014	1	1	1	1	2	1	30d	1	8
Maddox 2013	1	1	1	1	2	1	?	0	7
Mugnai 2017	1	1	1	1	2	1	3m	1	8

Murakawa 2014	1	1	1	1	2	1	?	0	7
Nagao 2014	1	1	1	1	2	1	30d	1	8
Nagao 2015	1	1	1	1	2	1	30d	1	8
Nakamura 2016	1	1	1	1	2	1	every 1-2m	1	8
Okishige 2016	1	1	1	1	2	1	3m	1	8
Okumura 2016	1	1	1	1	2	1	30d	1	8
Reynolds 2017	1	1	1	1	2	1	30d	1	8
Sawhney 2018	1	1	1	1	2	1	3m	1	8
Shah 2017	1	1	1	1	2	1	90d	1	8
Silva 2020	1	1	1	1	2	1	?	1	8
Snipelisky 2012	1	1	1	1	1	1	1w	0	6
Snipelisky 2014	1	1	1	1	2	1	2w	0	7
Somani 2014	1	1	1	1	2	1	3m	1	8
Stepanyan 2014	1	1	1	1	2	1	30d	1	8
Tao 2016	1	1	1	1	2	1	>2w	0	7
Tscholl 2017	1	1	1	1	2	1	3m	1	8
Yamaji 2013	1	1	1	1	2	1	90d	1	8
Yoshimura 2016	1	1	1	1	2	1	?	0	7

Table S2: Assessment of bias in randomized clinical trials using the Cochrane Collaboration's Tool

Author and date	Selection bias	Performance bias	Detection bias	Attrition bias	Reporting bias
Calkins 2017	Low	High	Low	Low	Low
Cappato 2015	Low	High	Low	Low	Low
Hohnloser 2019	Low	High	Low	Low	Low
Nogami 2019	Low	High	Low	Low	Low
Kirchhof 2018	Low	High	Low	Low	Low
Kuwahara 2016	Unclear	High	Unclear	Low	Low

Table S3: Detailed baseline characteristics

attached in separate file

Table S4: Results of Leave-One-Out analysis in the uninterrupted and minimally interrupted treatment group, major bleeding

Uninterrupted			
**Study omitted**	**Estimate**	**[95% Conf.**	**Interval]**
Calkins (2017)	.8285085	.5826633	1.178084
Kuwahara (2016)	.70015019	.5017345	.97703135
Cappato (2015)	.7170465	.51384121	1.000612
Kirchhof (2018)	.73073	.51143509	1.044055
Hohnloser (2019)	.73213845	.52392423	1.0230997
Tao (2016)	.71758837	.51423329	1.0013607
Okumura (2016)	.72280842	.51590115	1.0126978
Nagao (2015)	.70219427	.50168669	.98283815
Di Biase (2015)	.69703269	.49879888	.97404903
Nagao (2014)	.71033454	.50811899	.99302566
Lakkireddy (2014)	.71127838	.50328267	1.0052342
Maddox (2013)	.71646583	.51104468	1.0044587
Shah (2017)	.69423521	.49228233	.97903681
Dillier (2014)	.70765966	.50680751	.98811126
Reynolds (2017)	.70220912	.50088161	.98445946
Brink-Theo (2018)	.68643028	.48617244	.9691757
Kojima (2018)	.69709259	.49886537	.9740867
Sawhney (2018)	.62576568	.42784825	.91523731
Silva (2020)	.71843261	.51412034	1.0039389
**Combined**	.71115434	.51052718	.99062405
**Minimally interrupted**			
**Study omitted**	**Estimate**	**[95% Conf.**	**Interval]**
Nogami (2019)	.91484118	.44709727	1.871929
Yoshimura (2016)	.80268973	.39635247	1.6256005
Nakamura (2016)	.79406583	.40927204	1.5406392
Murakawa (2017)	.87228757	.39622873	1.9203191
Snipelisky (2014)	.82299459	.41198051	1.6440586
Ichiki (2013)	.59167141	.3523609	.99351269
Aso (2015)	.78619784	.39108863	1.5804781
Bassiouny (2013)	.8191613	.39272025	1.7086595
Kaseno (2012)	.84405941	.42510551	1.6759045
Lakkireddy (2012)	.66243851	.36774591	1.1932826
Okishige (2016)	.88764149	.4457401	1.7676387
Snipelisky (2012)	.79406583	.40927204	1.5406392
Tscholl (2017)	.7804774	.38645828	1.5762244
Reynolds (2017)	.73996371	.36227006	1.5114312
Yamaji (2013)	.82000279	.40933338	1.6426818
**Combined**	.79406583	.40927205	1.5406392

Table S5: Results of Leave-One-Out analysis in the RCT group, composite endpoint

**Study omitted**	**Estimate**	**[95% Conf.**	**Interval]**
Calkins (2017)	.44730592	.19718444	1.0146976
Cappato (2015)	.37303573	.16873363	.8247062
Hohnloser (2019)	.37921894	.16935676	.84913647
Kirchhof (2018)	.23474969	.11643645	.47328317
Kuwahara (2016)	.32601288	.162343	.65469044
Nogami (2019)	.38636476	.15422741	.96790653
**Combined**	.35497193	.17228599	.73137158

Figures S1.x: Baseline characteristics of the included studies

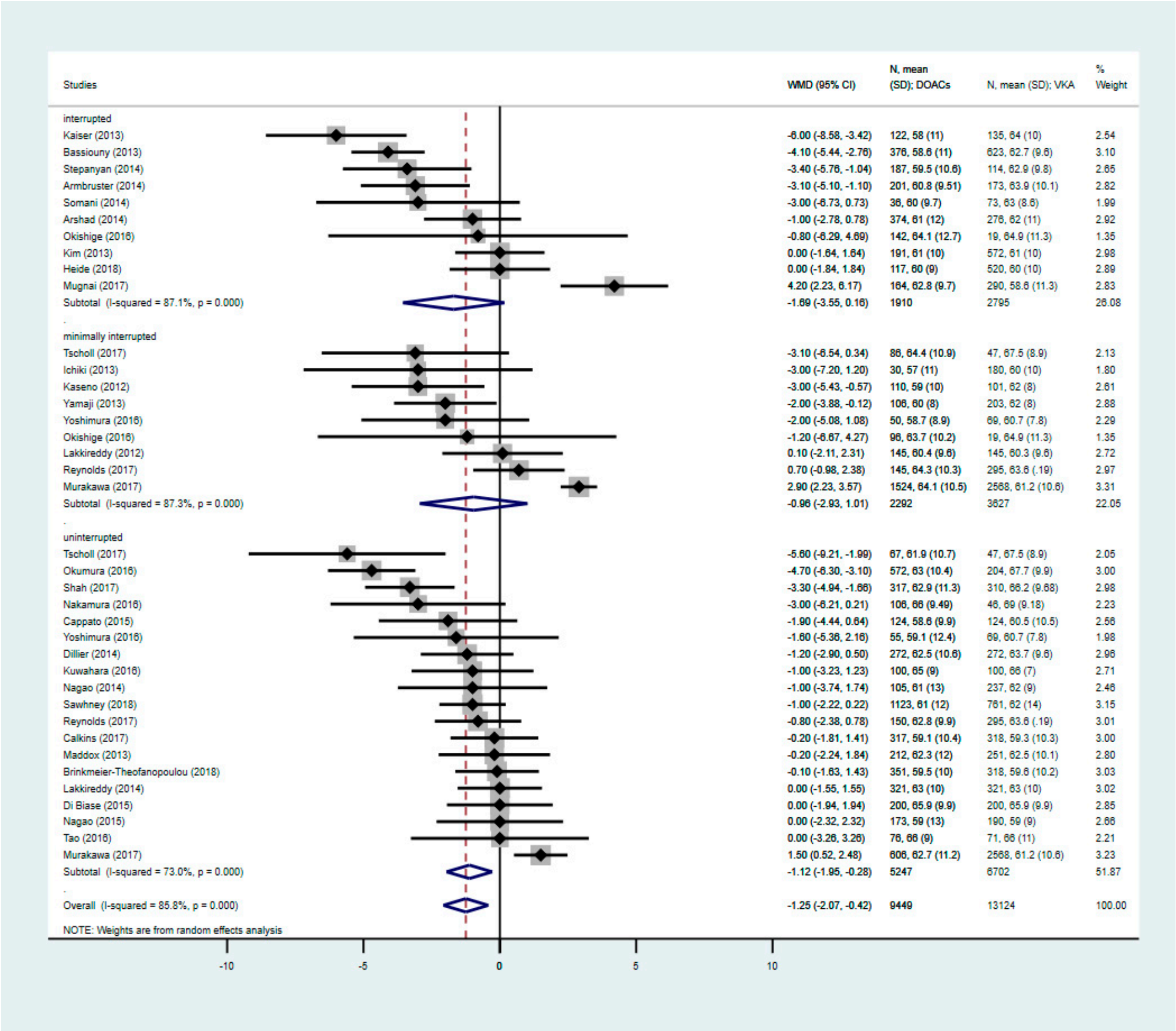

Figure S1.1 Age

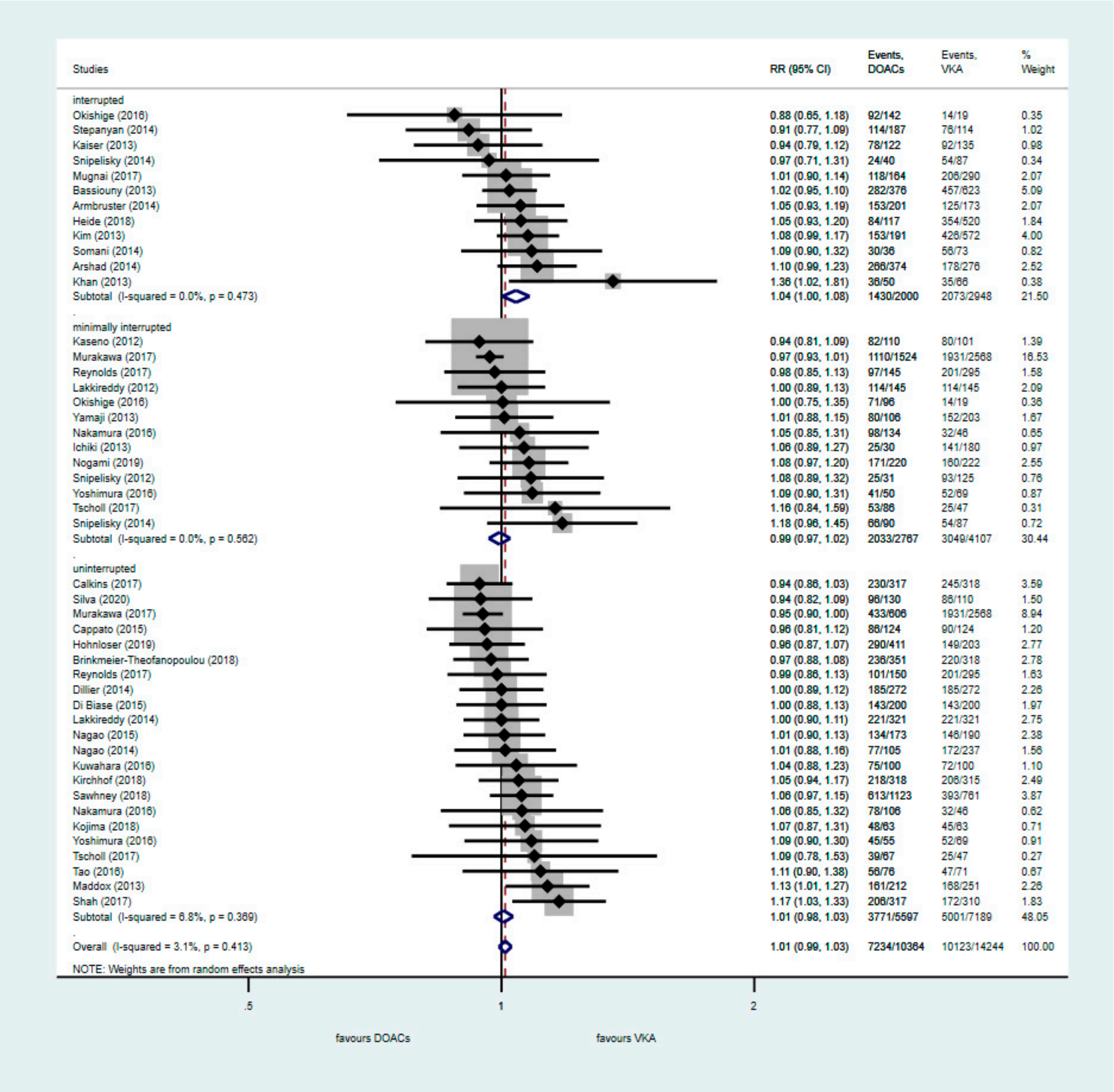

Figure S1.2 Males

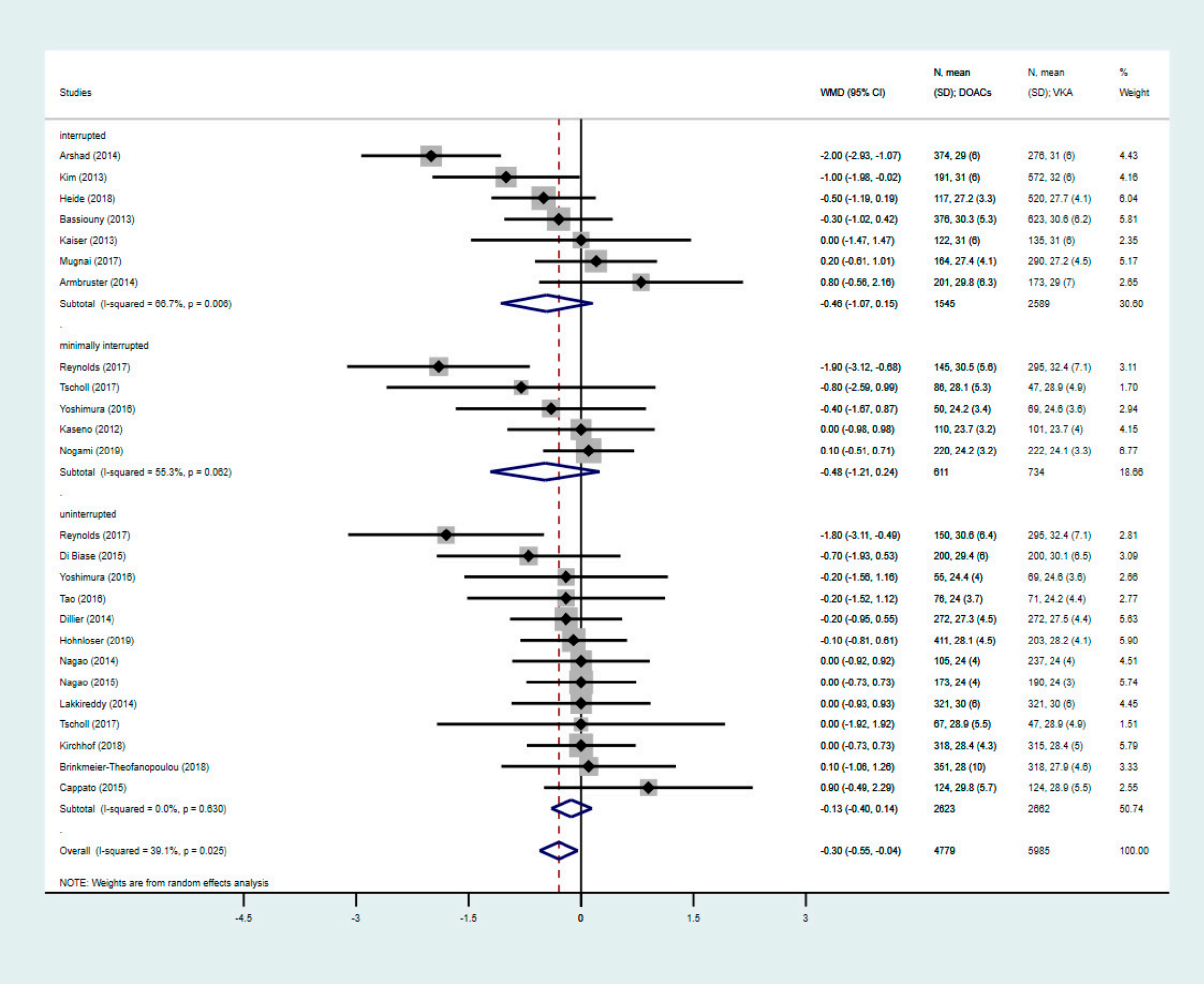

Figure S1.3 BMI

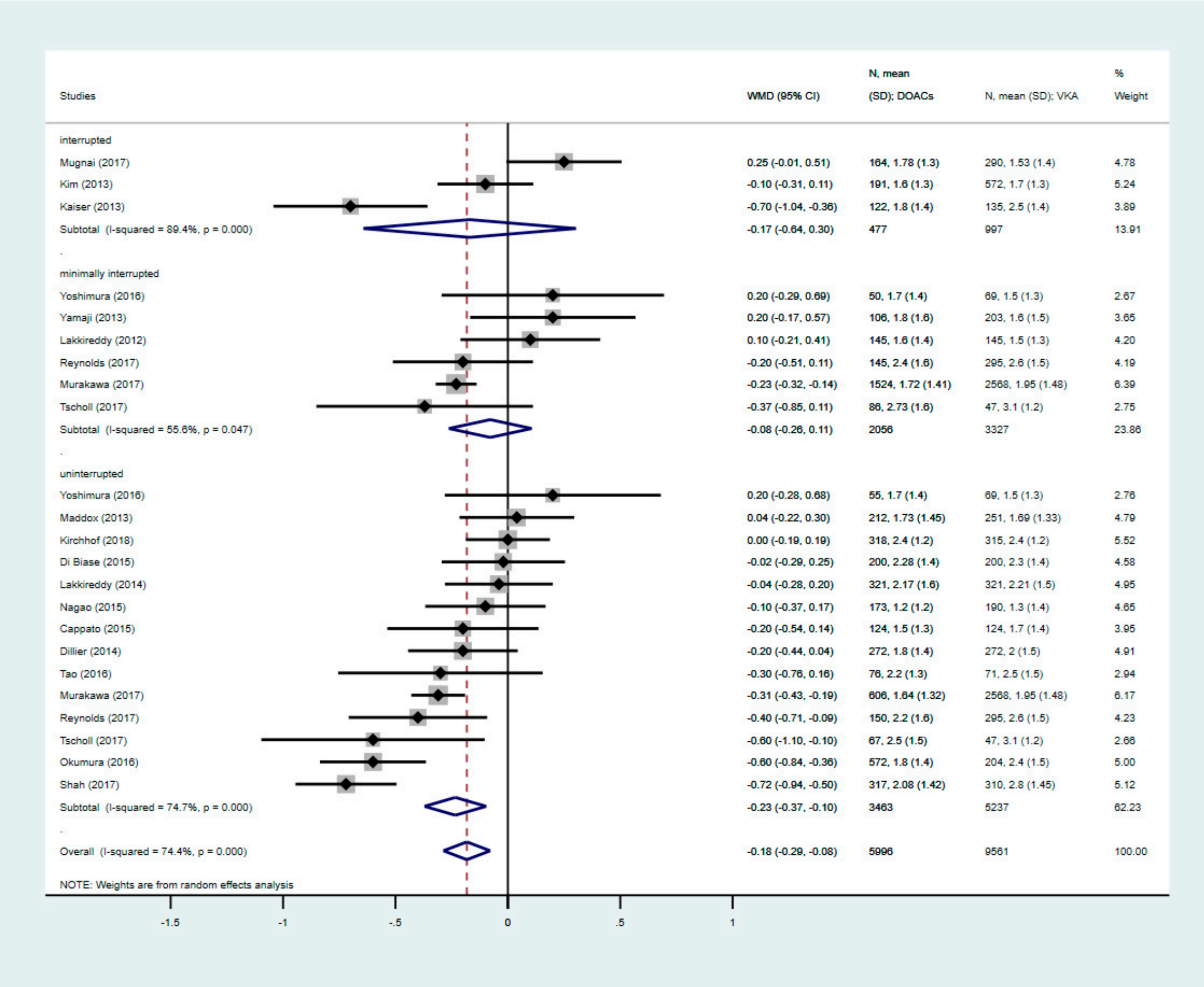

Figure S1.4 CHA_2DS_2-VASc

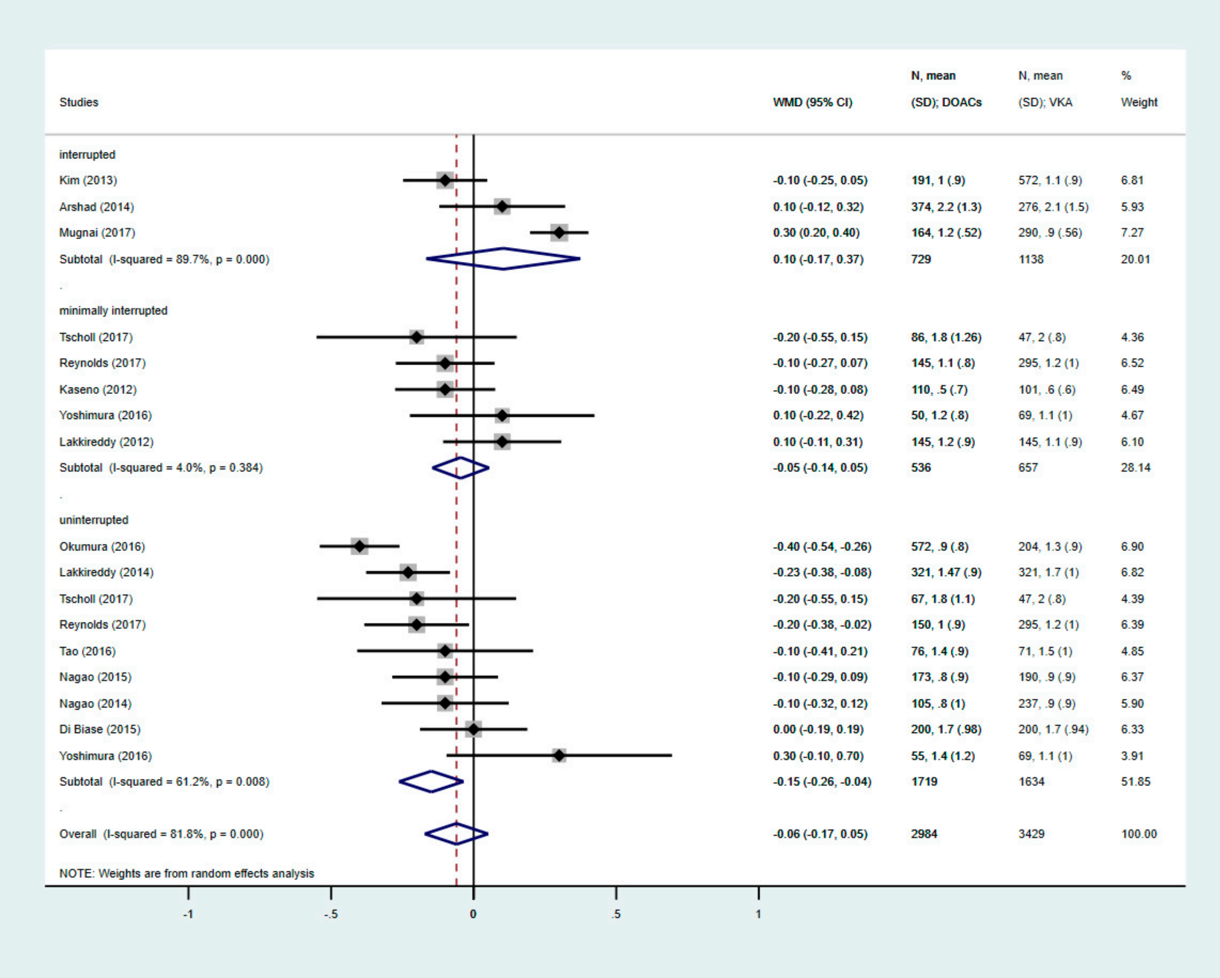

Figure S1.5 HASBLED

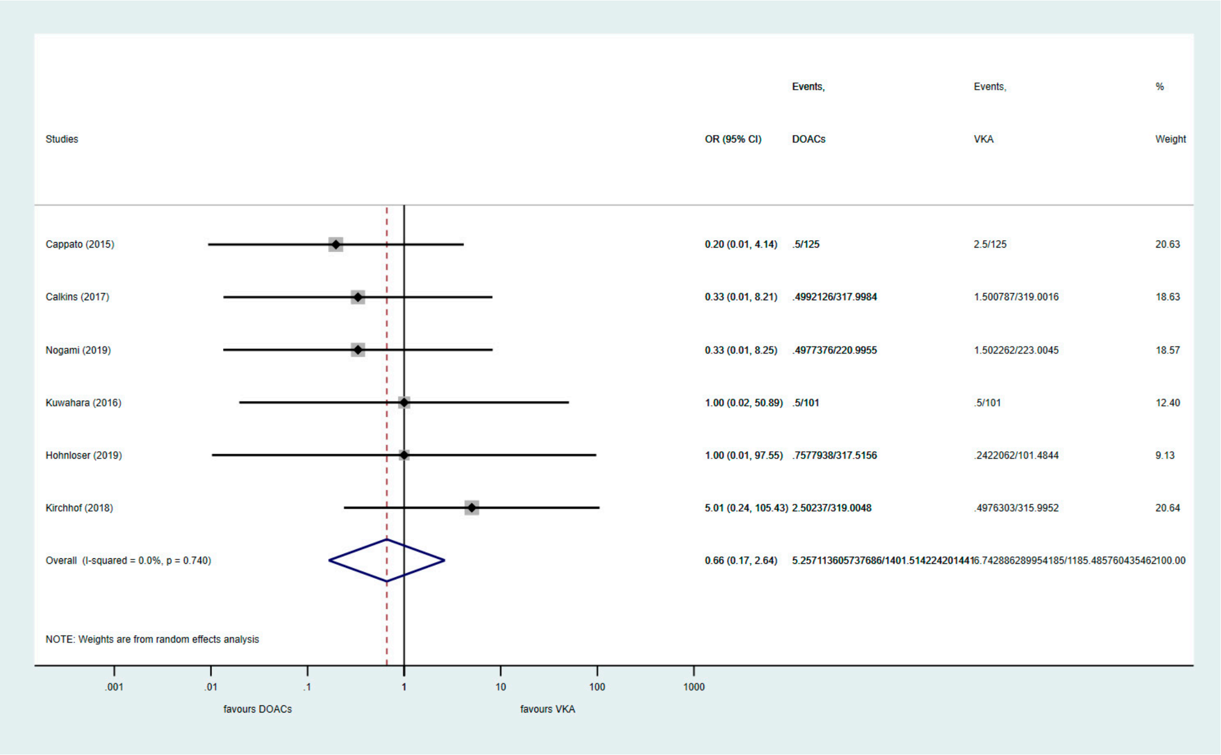

Figure S2. Outcome of RCTs: Stroke and TIA

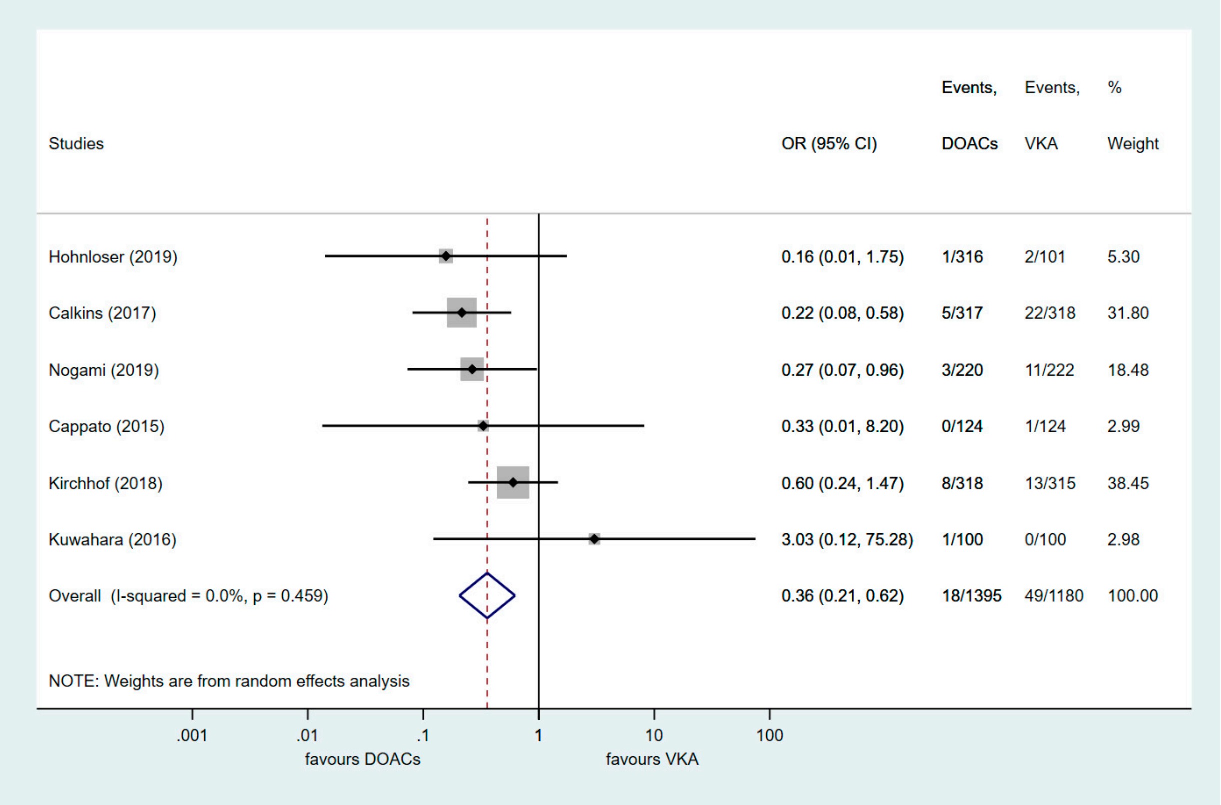

Figure S3. Outcome of RCTs: Major Bleeding

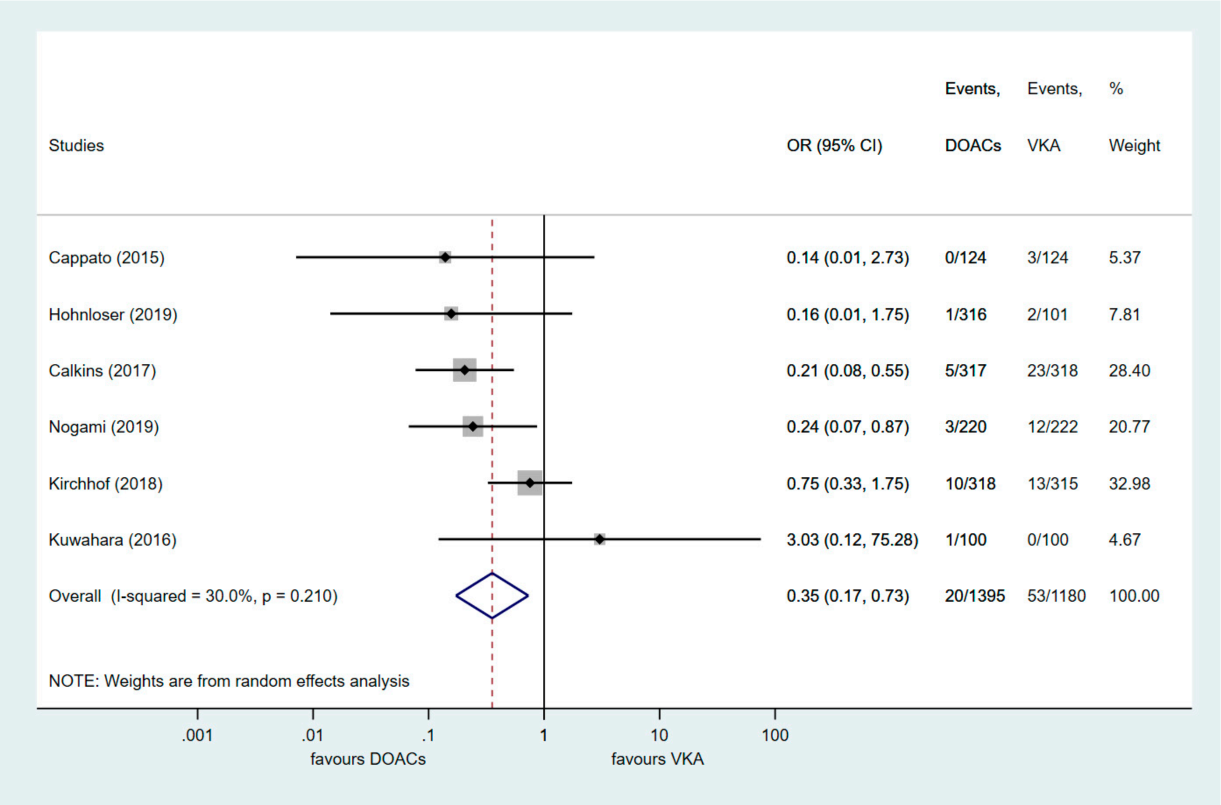

Figure S4. Composite endpoint of RCTs

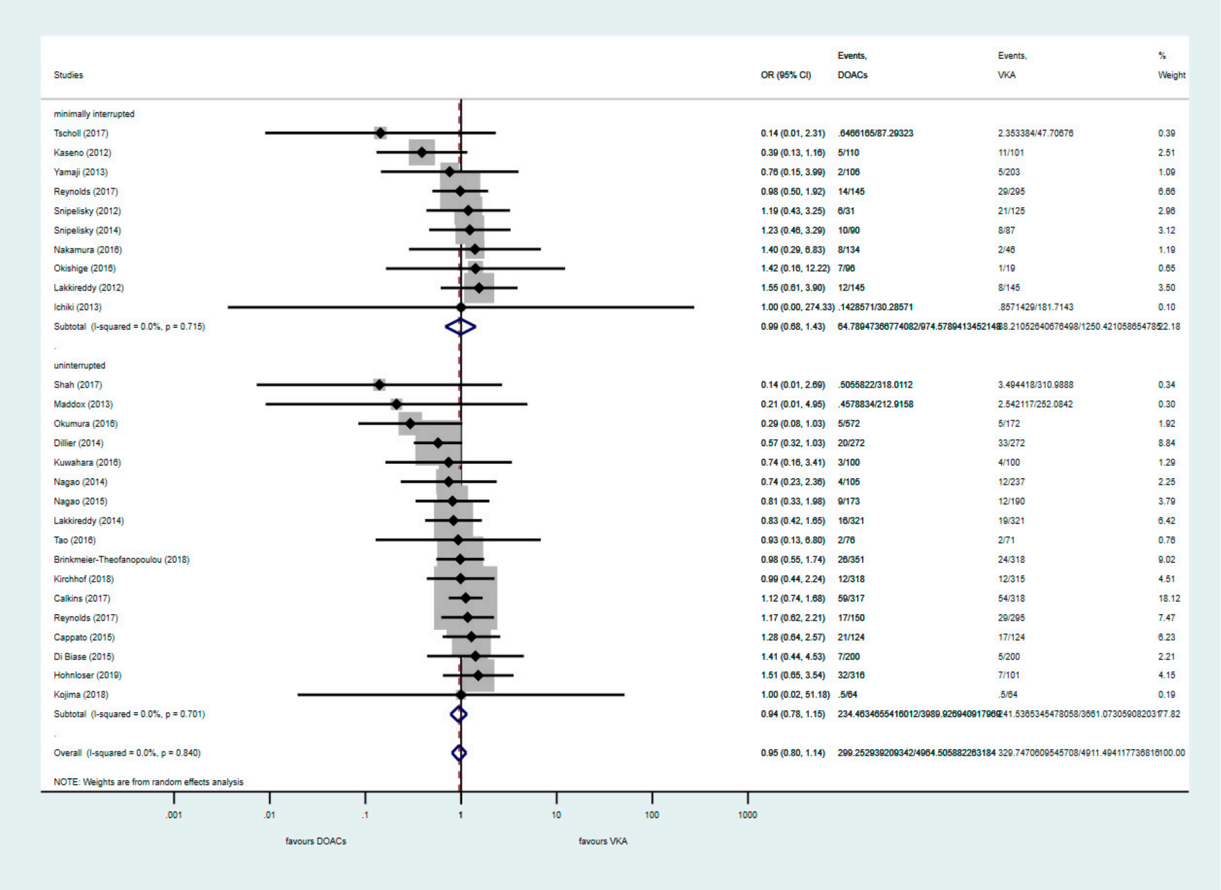

Figure S5: Minimally interrupted and Uninterrupted therapy outcomes: Minor bleeding

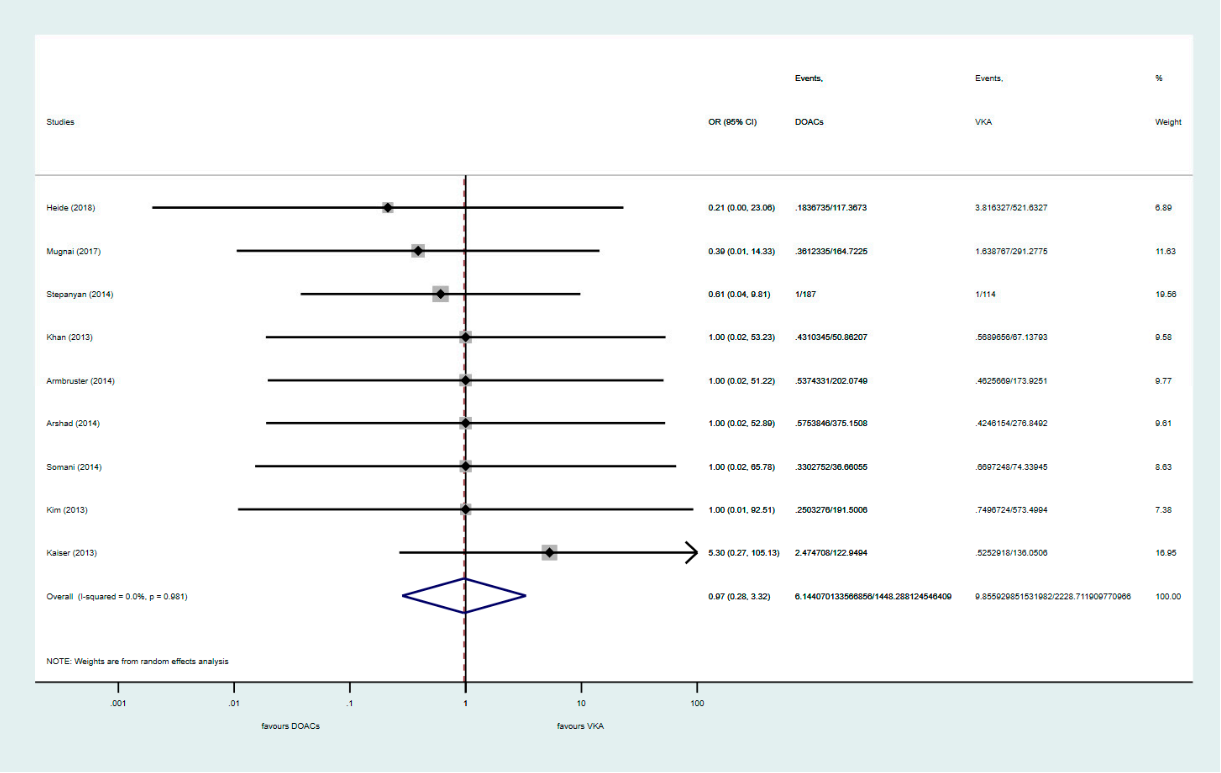

Figure S6: Interrupted therapy outcome: Stroke and TIA

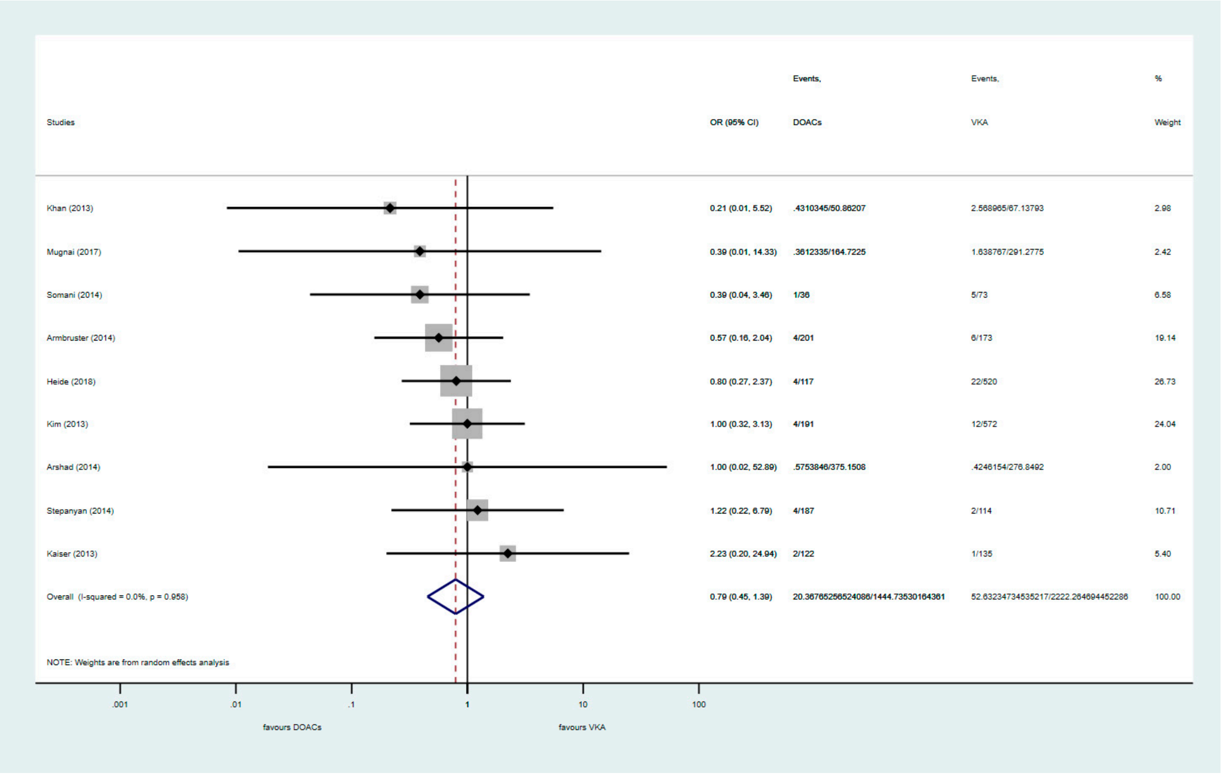

Figure S7: Interrupted therapy outcome: Major bleeding

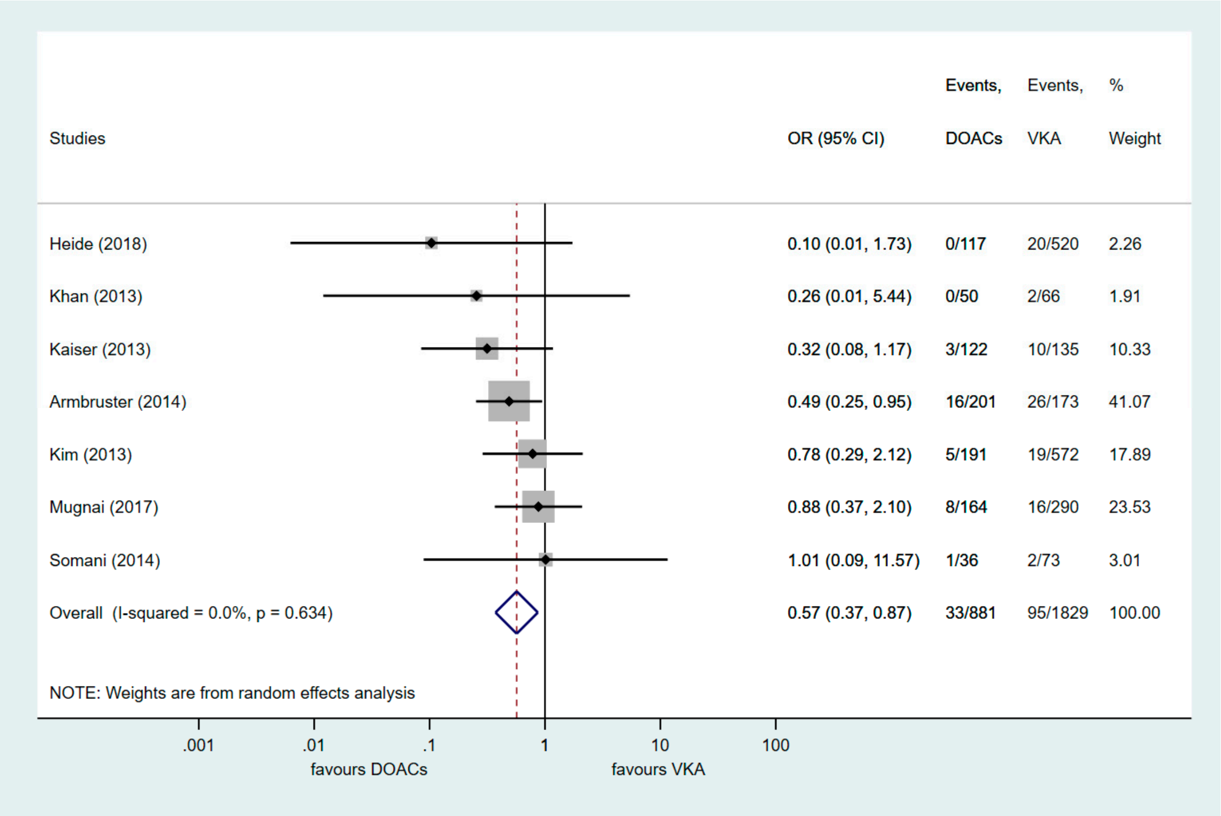

Figure S8: Interrupted therapy outcome: Minor bleeding

References of analyzed studies:

Randomized Controlled Trials:

Calkins H, Willems S, Gerstenfeld EP, Verma A, Schilling R, Hohnloser SH, et al. Uninterrupted Dabigatran versus Warfarin for Ablation in Atrial Fibrillation. N Engl J Med 2017; 376:1627-1636

Cappato R, Marchlinski FE, Hohnloser SH, Naccarelli GV, Xiang J, Wilber DJ, et al. Uninterrupted rivaroxaban vs. uninterrupted vitamin K antagonists for catheter ablation in non-valvular atrial fibrillation. Eur Heart J. 2015 Jul 21;36(28):1805-11.

Hohnloser SH, Camm J, Cappato R, Diener HC, Heidbüchel H, Mont L, et al. Uninterrupted edoxaban vs. Vitamin K antagonists for ablation of atrial fibrillation: the ELIMINATE-AF trial. European Heart Journal (2019) 00, 1–9.

Kirchhof P, Haeusler KG, Blank B, De Bono J, Callans D, Elvan A, et al. Apixaban in patients at risk of stroke undergoing atrial fibrillation ablation. Eur Heart J. 2018 Aug 21;39(32):2942-2955.

Kuwahara T, Abe M, Yamaki M, Fujieda H, Abe Y, Hashimoto K, et al. Apixaban versus Warfarin for the Prevention of Periprocedural Cerebral Thromboembolism in Atrial Fibrillation Ablation: Multicenter Prospective Randomized Study. J Cardiovasc Electrophysiol 2016;27:549-554

Nogami A, Harada T, Sekiguchi Y, Otani R, Yoshida Y, Yoshida K, et al. Safety and Efficacy of Minimally Interrupted Dabigatran vs Uninterrupted Warfarin Therapy in Adults Undergoing Atrial Fibrillation Catheter Ablation: A Randomized Clinical Trial. JAMA Netw Open. 2019 Apr 5;2(4):e191994.

Uninterrupted DOAC studies:

Brinkmeier-Theofanopoulou M, Tzamalis P, Wehrkamp-Richter S, Radzewitz A, Merkel M, Schymik G, et al. Periprocedural anticoagulation during left atrial ablation: interrupted and uninterrupted vitamin K-antagonists or uninterrupted novel anticoagulants. BMC Cardiovascular Disorders 2018 Apr 27;18(1):71.

Di Biase L, Lakkiredy D, Trivedi C, Deneke T, Martinek M, Mohanty S, et al. Feasibility and safety of uninterrupted periprocedural apixaban administration in patients undergoing radiofrequency catheter ablation for atrial fibrillation: Results from a multicenter study. Heart Rhythm. 2015 Jun;12(6):1162-8.

Dillier R, Ammar S, Hessling G, Kaess B, Pavaci H, Buiatti A, et al. Safety of Continuous Periprocedural Rivaroxaban for Patients Undergoing Left Atrial Catheter Ablation Procedures. Circ Arrhythm Electrophysiol. 2014;7:576-582.

Kojima T, Fujiu K, Fukuma N, Matsunaga H, Oshima T, Matsuda J, et al. Periprocedural Complications in Patients Undergoing Catheter Ablation of Atrial Fibrillation Without Discontinuation of a Vitamin K Antagonist and Direct Oral Anticoagulants. Circ J. 2018 May 25;82(6):1552-1557.

Lakkireddy D, Reddy YM, DiBiase L, Vallakati A, Mansour MC, Santangeli P, et al. Feasibility and Safety of Uninterrupted Rivaroxaban for Periprocedural Anticoagulation in Patients Undergoing Radiofrequency Ablation for Atrial Fibrillation. J Am Coll Cardiol 2014;63:982–8

Maddox W, Kay N, Yamada T, Osorio J, Doppalapudi H, Plumb VJ, et al. Dabigatran versus Warfarin Therapy for Uninterrupted Oral Anticoagulation During Atrial Fibrillation Ablation. J Cardiovasc Electrophysiol 2013;24:861-865

Nagao T, Inden Y, Shimano M, Fujita M, Yanagisawa S, Kato H, et al. Efficacy and Safety of Apixaban in the Patients Undergoing the Ablation of Atrial Fibrillation. Pacing Clin Electrophysiol. 2015 Feb;38(2):155-63.

Nagao T, Inden Y, Shimano M, Fujita M, Yanagisawa S, Kato H, et al. Feasibility and Safety of Uninterrupted Dabigatran Therapy in Patients Undergoing Ablation for Atrial Fibrillation. Intern Med 54: 1167-1173, 2015

Okumura K, Aonuma K, Kumagai K, Hirao K, Inoue K, Kimura M, et al. Efficacy and Safety of Rivaroxaban and Warfarin in the Perioperative Period of Catheter Ablation for Atrial Fibrillation. Circ J 2016; 80: 2295–2301

Reynolds MR, Allison JS, Natale A, Weisberg IL, Ellenbogen KA, Richards M, et al. A Prospective Randomized Trial of Apixaban Dosing During Atrial Fibrillation Ablation. JACC Clin Electrophysiol. 2018 May;4(5):580-588.

Sawhney V, Shaukat M, Volkova E, Jones N, Providencia R, Honarbakhsh S, et al. Catheter ablation for atrial fibrillation on uninterrupted direct oral anticoagulants: A safe approach. Pacing Clin Electrophysiol. 2018 May 16. doi: 10.1111/pace.13370.

Shah RR, Pillai A, Schafer P, Meggo D, McElderry T, Plumb V, et al. Safety and Efficacy of Uninterrupted Apixaban Therapy Versus Warfarin During Atrial Fibrillation Ablation. Am J Cardiol. 2017 Aug 1;120(3):404-407.

Silva MA, Futuro GMC, Merçon ES, Vasconcelos D, Agrizzi RS, Elias Neto J, et al. Safety of Catheter Ablation of Atrial Fibrillation Under Uninterrupted Rivaroxaban Use. Arq Bras Cardiol. 2020 Mar;114(3):435-442.

Tao S, Otomo K, Ono Y, Osaka Y, Hirao T, Koura K, et al. Efficacy and safety of uninterrupted rivaroxaban taken preoperatively for radiofrequency catheter ablation of atrial fibrillation compared to uninterrupted warfarin. J Interv Card Electrophysiol (2017) 48:167–175

Minimally interrupted DOAC studies:

Aso T, Nakamura T, Nishihara M, et al. The incidence of silent cerebral thromboembolism in catheter ablation for atrial fibrillation using novel oral anticoagulants versus therapeutic warfarin. Conference abstract of the European Heart Rhythm Association Europace 2015 Conference, 2015, London, UK. EP Europace 2015;17(3):iii234-iii236

Bassiouny M, Saliba W, Rickard J, Shao M, Sey A, Diab M, et al. Use of dabigatran for periprocedural anticoagulation in patients undergoing catheter ablation for atrial fibrillation. Circ Arrhythm Electrophysiol 2013;6(3):460–466.

Ichiki H, Oketani N, Sanemasa I, Iriki Y, Okui H, Maenosono R, et al. The Incidence of Asymptomatic Cerebral Microthromboembolism after Atrial Fibrillation Ablation: Comparison of Warfarin and Dabigatran. Pacing Clin Electrophysiol. 2013 Nov;36(11):1328-35.

Kaseno K, Naito S, Nakamura K, Sakamoto T, Sasaki T, Tsukada N, et al. Efficacy and Safety of Periprocedural Dabigatran in Patients Undergoing Catheter Ablation of Atrial Fibrillation. Circ J 2012; 76:2337–2342.

Lakkireddy D, Reddy YM, DiBiase L, Vanga SR, Santangeli P, Swarup V, et al. Feasibility and Safety of Dabigatran Versus Warfarin for Periprocedural Anticoagulation in Patients Undergoing Radiofrequency Ablation for Atrial Fibrillation. J Am Coll Cardiol 2012;59:1168–74

Murakawa Y, Nogami A, Shoda M, Inoue K, Naito S, Kumagai K, et al. Report of periprocedural oral anticoagulants in catheter ablation for atrial fibrillation: The Japanese Catheter Ablation Registry of Atrial Fibrillation (J-CARAF). J Arrhythm. 2017 Jun;33(3):172-176.

Nakamura K, Naito S, Sasaki T, Minami K, Take Y, Goto E, et al. Silent Cerebral Ischemic Lesions After Catheter Ablation of Atrial Fibrillation in Patients on 5 Types of Periprocedural Oral Anticoagulation. Circ J 2016; 80: 870–877

Okishige K, Nakamura T, Aoyagi H, Kawaguchi N, Yamashita M, Kurabayashi M, et al. Comparative study of hemorrhagic and ischemic complications among anticoagulants in patients undergoing cryoballoon ablation for atrial fibrillation. J Cardiol. 2017 Jan;69(1):11-15.

Snipelsky D, Kauffman C, Prussak K, Johns G, Venkatachalam K, Kusumoto F. A comparison of bleeding complications post-ablation between warfarin and dabigatran. J Interv Card Electrophysiol. 2012 Oct;35(1):29-33.

Snipelsky D,Ray JC, Ung R, Duart M, Kauffman C, Kusumoto F. A comparison of bleeding complications between warfarin, dabigatran, and rivaroxaban in patients undergoing cryoballoon ablation. J Interv Card Electrophysiol. 2014 Dec;41(3):231-6.

Tscholl V, Lsharaf AKA, Lin T, Bellmann B, Nagel P, Lenz K, et al. Apixaban, rivaroxaban, and dabigatran use in patients undergoing catheter ablation for atrial fibrillation using the second-generation cryoballoon. Clin Cardiol. 2017 Nov;40(11):1095-1099.

Yamaji H, Murakami T, Hina K, Higashiya S, Kawamura H, Murakami M, et al. Usefulness of Dabigatran Etexilate as Periprocedural Anticoagulation Therapy for Atrial Fibrillation Ablation. Clin Drug Investig. 2013 Jun;33(6):409-18.

Yoshimura A, Iriki Y, Ichiki H, Oketani N, Okui H, Maenosono R, et al. Evaluation of safety and efficacy of periprocedural use of rivaroxaban and apixaban in catheter ablation for atrial fibrillation. J Cardiol. 2017 Jan;69(1):228-235.

Interrupted DOAC studies:

Armbruster HL, Lindsley JP, Moranville MP, Habibi M, Khurram IM, Spragg DD, et al. Safety of novel oral anticoagulants compared with uninterrupted warfarin for catheter ablation of atrial fibrillation. Ann Pharmacother 2015;49(3):278–284.

Arshad A, Johnson CK, Mittal S, Buch E, Hamam I, Tran T, et al. Comparative Safety of Periablation Anticoagulation Strategies for Atrial Fibrillation: Data from a Large Multicenter Study. Pacing Clin Electrophysiol. 2014 June ; 37(6): 665–673.

Heide J, Vroegh CJ, Bhagwandien RE, Wijchers SA, Szili-Torok T, Zijlstra F, et al. Minimally interrupted novel oral anticoagulant versus uninterrupted vitamin K antagonist during atrial fibrillation ablation. J Interv Card Electrophysiol. 2018 Dec;53(3):341-346.

Kaiser DW, Streur MM, Nagarakanti R, Whalen SP, Ellis CR. Continuous warfarin versus periprocedural dabigatran to reduce stroke and systemic embolism in patients undergoing catheter ablation for atrial fibrillation or left atrial flutter. J Interv Card Electrophysiol. 2013 Sep;37(3):241-7.

Khan S, Duggal M, Dunskis P, et al. Periprocedural Dabigatran in patients undergoing catheter ablation for atrial fibrillation. Moderated Poster Presentation at ACC.13, March 11, 2013, San Fransisco, CA, USA, JACC 2013;61(10)

Kim JS, She F, Jongnarangsin K, Chugh A, Latchamsetty R, Ghanbari H, et al. Dabigatran vs warfarin for radiofrequency catheter ablation of atrial fibrillation. Heart Rhythm 2013;10:483–489

Mugnai G, de Asmundis C, Iacopino S, Stroker E, Longobardi M, De Regibus V, et al. Comparison of the Incidences of Complications After Second-Generation Cryoballoon Ablation of Atrial Fibrillation Using Vitamin K Antagonists. Am J Cardiol. 2017 Jul 15;120(2):223-229.

Somani R, Mohajer K, Haley C, Simpson CS, Abdollah H, Baranchuk A, et al. The Peri-procedural Use of Dabigatran in Patients Undergoing Left Atrial Ablation for Atrial Fibrillation. Cardiovascular Ther. 32 (2014) 198–201.

Stepanyan G, Badhwar N, Lee RJ, Marcus GM, Lee BK, Tseng ZH, et al. Safety of new oral anticoagulants for patients undergoing atrial fibrillation ablation. J Interv Card Electrophysiol (2014) 40:33–38
